# Scaling up impact of malaria control programmes: a tale of events in Sub-Saharan Africa and People’s Republic of China

**DOI:** 10.1186/2049-9957-1-7

**Published:** 2012-11-01

**Authors:** Ernest Tambo, Ahmed Adebowale Adedeji, Fang Huang, Jun-Hu Chen, Shui-Sen Zhou, Ling-Hua Tang

**Affiliations:** 1 National Institute of Parasitic Disease, Chinese Center for Disease Control and Prevention, WHO Collaborating Centre on Malaria, Schisostomiasis and Filariasis, Key Laboratory of Parasite and Vector Biology, Ministry of Health, 207 Rui Jin Er Rd, Shanghai, 200025, People’s Republic of China; 2 School of Medicine & Pharmacy, Houdegbe North American University PK10, Route de Porto-Novo, 06 BP 2080, Cotonou, République du Bénin; 3 Department of Pharmacology and Toxicology, Kampala International University Western Campus, P.O.Box 71, Ishaka, Bushenyi, Uganda

**Keywords:** malaria, funding, scaling up, interventions, health system, Sub-Sahara Africa, People’s Republic of China

## Abstract

This review aims at providing synthetic information with scientific evidence on the trends in the malaria events from 1960 to 2011, with the hope that it will help policy makers to take informed decisions on public health issues and intervention designs on malaria control towards elimination in both Sub-Sahara Africa and in the People’s Republic of China by highlighting the achievements, progress and challenges in research on moving malaria from epidemic status towards elimination. Our findings showed that since 1960, malaria control programmes in most countries have been disjointed and not harmonized. Interestingly, during the last decade, the causal factors of the unprecedented and substantial decline in malaria morbidity and mortality rates in most vulnerable groups in these endemic areas are multifaceted, including not only the spread of malaria and its related effects but also political and financial willingness, commitment and funding by governments and international donors. The benefits of scaling up the impact of malaria coverage interventions, improvement of health system approaches and sustained commitment of stakeholders are highlighted, although considerable efforts are still necessary in Sub-Sahara Africa. Furthermore, novel integrated control strategies aiming at moving malaria from epidemic status to control towards elimination, require solid research priorities both for sustainability of the most efficient existing tools and intervention coverage, and in gaining more insights in the understanding of the epidemiology, pathogenesis, vector dynamics, and socioeconomic aspects of the disease. In conclusion, political commitment and financial investment of stakeholders in sustaining the scaling up impact of malaria control interventions, networking between African and Chinese scientists, and their Western partners are urgently needed in upholding the recent gains, and in translating lessons learnt from the Chinese malaria control achievements and successes into practical interventions in malaria endemic countries in Africa and elsewhere.

## Multilingual abstracts

Please see Additional file 1 for translations of the abstract into the six official working languages of the United Nations.

## Review

The review highlights malaria events, achievements and scaling up impact with scientific evidence in moving malaria from epidemic status towards sustained control and elimination from 1960–2011. The unprecedented and substantial reduction in malaria incidence and consequently mortality rates, at varied degrees across African countries and People’s Republic of China (P.R.China) are very encouraging, although the gains are still fragile. Increased political commitment and available financial resources collectively from governments and various stakeholders are paramount in sustaining the scaling up innovative and integrated malaria control interventions, and health system strengthening to turn the tide against the malaria public health and its related effects in Sub-Saharan Africa (SS Africa) and elsewhere.

## Background

There has been improvement in the health situation in most African countries since 1960 and malaria in particular is decreasing over time in SS Africa; where the global burden of the disease is significantly approximately 90% and P.R. China accounts for less than 10%. The recent statistics showing reductions in malaria deaths are very encouraging, but the situation is fragile and malaria should not be allowed to resurge [1-3]. Malaria is a vector-borne disease caused by protozoan parasites, i.e. *Plasmodium falciparum, P. vivax, P. malariae, P. ovale,* or *P. knowlesi* that completes a complex cycle of development alternating between human hosts and mosquitoes of the genus *Anopheles*[4]*.* The disease emerged as a worldwide epidemic in the 1960s when global malaria eradication was abandoned [5,6]. Consequently, the emergence of insecticide resistant and drug resistant parasite strains and vectors were greatly fuelled by poverty, poor economy, political instability, poor health infrastructure and equipment, deficiencies in health systems and policy particularly in Africa [4,7,8]. The public burden and impact of this disease on human health, productivity, loss of work efficiency and time in malarial regions have been devastating [9].

To strengthen the efforts towards the fight against malaria, the World Health Assembly passed a resolution on controlling malaria in Africa in May, 1996 and this was closely followed by declarations of the Organization of African Unity(OAU) on malaria in Harare, Zimbabwe in 1997 and Ouagadougou, Burkina Faso in 1998 [10]. In 1996, the African Regional Office of the World Health Organization (WHO) became increasingly attentive to malaria and launched the African Initiative for Malaria Control (AIM). The AIM contributed $9 million in 1997 and 1998, for accelerated implementation of malaria control activities in 10 countries in the region, and provided the foundation for the eventual launch of Roll Back Malaria (RBM) in 1998 [11]. The African Heads of States Summit to Roll Back Malaria was held in April 2000 in Abuja, Nigeria, where they set the goal for reducing malaria deaths to half by the year 2010. The diverse array of meetings, programmes, and activities are testimony to the growing recognition of the regional and global nature of the threat posed by malaria [11-13]. Strong political commitment, financial support and partnerships are important to bring about the current desired changes. Malaria re-emerged as a major international health issue in the 1990s, despite the global malaria control strategy adopted in 1992, RBM in 1998, Abuja Declaration in 2000 and strong political commitment and partnership respectively. In the face of these, malaria is still prevalent in 106 countries of the tropical and sub-tropical world, with 51 countries in the African continent bearing the highest burden of cases and deaths [2,9,14-16]*.* No experts from SS Africa were involved in the Global Malaria Eradication Program (GMEP) due to the lack of African expertise at that time, the majority of African countries were driven by representatives of western countries taking decisions on their behalf in international forums, in addition to socio-cultural beliefs, a high degree of malaria endemicity in the region combined with lack or weak health policy and infrastructures, and others factors.

In P.R. China, mass patriotic health campaigns and mass mobilization approaches on preventive efforts in 1960s led to eradication of cholera, plague, scarlet fever, typhoid and syphilis with a considerable reduction of the burden of infectious diseases [4,17,18]. Within the same period, the Chinese government had implemented birth control measures and 40-45% of the rural Chinese populations were covered by cooperative medical systems. Efforts at increasing the acute shortage of medical personnel and facilities during1984 -1986 and the economic boom of 1987 in P.R. China led to fundamental urban and rural healthcare reforms systems through provision of preventive and curative interventions [17,18]. Recently, P.R. China has launched a national malaria elimination campaign (NMEP) as the cornerstone of a successful and intense surveillance and monitoring of vectors foci, disease management and potential outbreaks [17].

The design and implementation of policies for malaria control and elimination in tropical and sub-tropical areas have been mitigated by the consequences of political and financial agenda and achievements. A clear understanding of the interrelations between malaria and the cross-effects of targeting policies and financing on health systems are of valuable importance in sustainability. There are several challenges linked with the scaling up of malaria control interventions with no clear consensus and road map on how existing tools should be efficiently and continuously deployed in achieving targeted goals and eventual elimination, although several recent publications have targeting these issues [3,19-22]

In this review, therefore, we aimed at providing synthetic information with scientific evidence of scaling up impact of malaria control interventions on the trends of malaria events from 1960 to 2011, with the hope that it will help stakeholders, and policy-makers to take informed decisions on public health issues and intervention designs on malaria control towards elimination in SS Africa and P.R. China.

## Methodology

### Search strategy

A systematic search was conducted for articles published from January 1960 to December 2011 in PubMed/MEDLINE (OVID) (Originally Publius Ovidius Naso), Embase (OVID) Web of Knowledge, Scopus, and the WHO’s WHOLIS and regional office databases and CAB Direct databases using terms for malaria events and 49 target countries (48 African countries and P.R. China). The references were collated and categorized according to malaria, *Plasmodium* species, and whether they contained original or derivative data. The search was limited to studies with P.R. China and SS Africa medical subject headings (MeSH) term, involving interventions, epidemiologic and studies on malaria trends of events. All records resulting from these searches were screened, and full-text articles were assessed if the reference appeared to describe or allude to a malaria epidemic, control towards elimination event.

### Inclusion criteria

Publications relating to malaria in these countries within the time frame of January, 1960 to December, 2011 were assessed. Randomized controlled surveys, controlled before and after, uncontrolled before and after, interrupted time series, and cohort and case control studies were included. We assessed risk of bias for included studies but did not exclude studies on this basis. Accordingly, any report of an increase or decrease in malaria incidence or prevalence in assessed articles was included in analysis and included those published in English, French, or Chinese, regardless of article type or quality. Also national malaria strategic plans, malaria programme performance reviews and country led successful funding applications to the Global Fund to Fight AIDS, Tuberculosis and Malaria (GFMAT) on malaria were reviewed to explore how incidence and mortality trends, socio-economical and health reforms can be used in planning and decision making for malaria control and elimination in SS Africa and P.R. China.

### Evaluation of publications

Publication type for each article was derived from the PubMed/Medline, database and articles were categorized relevant to malaria research based on these publication types: i) political and financial analysis, ii) malaria health system reforms, iii) malaria incidence and prevalence and trends in interventions (vector/ parasite control). Malaria-related papers in which the *Plasmodium* species was not defined were categorized as "neither". Publications not meeting the inclusion criteria relevance for malaria were excluded.

### Data analysis

Data analysis were processed using Excel (Microsoft, WA, USA), and SPSS 13.0 was also used to compute the statistical comparisons, percentages and corresponding 95% confidence intervals (95% CI) were calculated using Wilson's method. The scaling up impact was evaluated as cumulative reduction in morbidity and mortality rates, and increasing life expectancy in vulnerable groups to the implemented malaria control interventions over time.

## Results

The database searches returned 2,171 articles, and 340 additional records were identified from hand-searching reference lists, producing a total of 883 (40.67%) unique records screened after removal of duplicates. Overall, 131 (14.83%) were published during 1960 to1990 (30 years) compared to an increase of approximately 6 times, 782 (85.16%) during 1990 to 2011(21 years). Of these, 89 described the trend of malaria events that were included and assessed from SS Africa and P.R. China during 1960–2011. Our findings showed three increasing areas of interest. First, the political and financial commitment and investments through scaling up of malaria intervention coverage programmes: (indoor residual spraying (IRS), insecticide-treated mosquito nets (ITNs), long lasting insecticides treated nets (LLINs), and intermittent preventive treatment during pregnancy (IPTp). Second, prompt and effective malaria case management with antimalarial drugs mainly the artemisinin based combination therapies (ACTs). Third, strengthening health system performance through increasing capacity building and delivery of malaria interventions, sustainability and universal coverage have brought about a dramatic health impact with short and long-term benefits. Interestingly, a substantial reduction in morbidity rate as well as on average more than a 20-58% mortality rate decrease in all vulnerable groups in most of SS Africa countries compared to 97.8% in P.R. China during the last decade.

### Malaria political and financial achievements from epidemic to control and elimination

Our findings showed that since the independence of the majority of African countries around the 1960's with limited capacities in malaria control and in P.R. China, political and financial commitments and strategies have permitted the achievements of essential milestones, moving malaria from epidemic towards control and elimination [1,14,23-27]. In the last two decades, control towards malaria elimination has been on the political agenda of several of the world’s wealthiest countries and funds have become available from the GFMAT, The US President’s Malaria Initiative (PMI), the World Bank, WHO and bilateral donors that are all financial sources for the fight against malaria. The RBM Partnership, coordinating the global fight against malaria, and major donor foundations, such as the Bill and Melinda Gates Foundation, National Institute of Health (NIH), The Coordination, Rationalization and Integration of antimalarial drug discovery and Development (CRIMALDDI), The Rockefeller Foundation, The Wellcome Trust, The ExxonMobil Foundation, USAID from the American People, The Coalition of Global Businesses have greatly increased financial support for malaria research and development as well as interventional approaches.

When analyzing on the basis of incidence and prevalence rates, the African malaria situation from 1960–2000 is comparable to that of P.R. China between 1960–1980 [4-7,9-29]. The scaling up of malaria coverage interventions across endemic areas testified the political and financial commitment of governments and stakeholders in achieving the millennium development goals (MDGs). Most African countries that have successfully implemented health policies, witnessed an improved and sustained nationwide coverage of malaria control measures and consequently documented a substantial decline in the morbidity and mortality rates amongst the population at risk, for example South Africa, Zanzibar, Gambia, Senegal, Tanzania, Kenya, Ghana and Cameroon) [3,22,30-32].

### Comparison of the trends of malaria morbidity and mortality rates from 1960–2011

Our findings showed that both P.R. China and Africa are located in the tropical and sub-tropics with optimal climatic and environmental conditions for the reproduction and development of *Anopheles* species. *P. falciparum* and *P. vivax* were shown to be major causative agents of malaria, respectively, having different degrees of virulence and similar disease pathophysiology. *P. vivax* accounts for 80-90% of malaria cases in the Middle East, Asia, and the Western Pacific tropical regions, 10-15% in Central and South America and less than 2% in Northern African countries [4,16,27,30]. Although the overall burden of malaria is higher in Africa than in P.R. China, there is increasing evidence that the overall burden, economic impact, and severity of disease have been underestimated [4,15-27,30-33].

Malaria public health burden during the 1960s-1970s was characterized by an upsurge in terms of malaria incidence and mortality rate in P.R. China as a result of increasing population demography and lack of adequate health infrastructure to cater for massive remotely located rural populations. Our findings showed that malaria publications from Africa from 1980–2000 are similar to those from P.R. China between 1960-1980s, which were characterized by a high death toll amongst the risky groups including children under the age of 5 years old, pregnant women and travelers [7-9,34-36]. Several reasons contributed to the huge toll of mortality including a higher degree of endemicity, post-independence instability in most African countries, lack of health infrastructures and resources, poor understanding of the disease and ecology, inability of “naive” leaders to generate income and/or to implement efficient healthcare reforms policy [9,12-19,28,29].

The era of 1970s-1980s was marked by a significant reduction in the infectious diseases in P.R. China including a drastic fall in malaria incidence (5,000 ‰ to 500 ‰), as a result of mass patriotic and mobilization health campaigns on prevention and implementation of birth control in the early 1970s. The ravage of malaria in Africa was increasingly higher with poverty related effects on the households, community and African countries [6,18-27,30-37]. With the structural adjustment plan proposed to African countries and implemented with the financial support of the International Monetary Fund, and The World Bank, part of the funds were allocated into the health sector but several factors contributed to the ineffectiveness of the plans. These included lack of political commitment, inadequate management and lack of much needed infrastructure in rural areas and difficult accessibility and availability of drugs, as well as lack of qualified medical personnel, with chronic pressure mounted on a few healthcare community workers has remained a huge challenge in some countries [25-27,30-38]. Most African countries faced the sorrowful period with alarming collision between the vicious cycle of malaria and poverty, and the impact of the Structural Adjusted Plan of the International Monetary Fund implemented in these countries. During the period 1980 – 1986, P.R. China registered an increase in morbidity rate to 500 ‰, reduction of life expectancy (less than 4 years) due to malaria, resulting from global economic crisis, dread shortage of health personnel and a weakened Chinese rural cooperative medical system. However, following by the P.R. China economic boom after 1987, there was a significant sharp drop of malaria incidence from 500 ‰ to 9.2 ‰ in 1990. This was thought to be brought about by the tremendous fundamental health system reforms, characterized by increasing support to collective welfare systems, provision of adequate preventive and curative health intervention packages through healthcare decentralization, primary healthcare reforms in 2005 and the basic healthcare with insurance schemes [17-27,30-36]. On the basis of these analyses of the trend of events, we came to the general conclusion that translations of national policy into innovative control strategies are imperative in strengthening the healthcare systems and actions to tackle the persistent burden of infectious diseases in most endemic countries.

**Figure 1:**
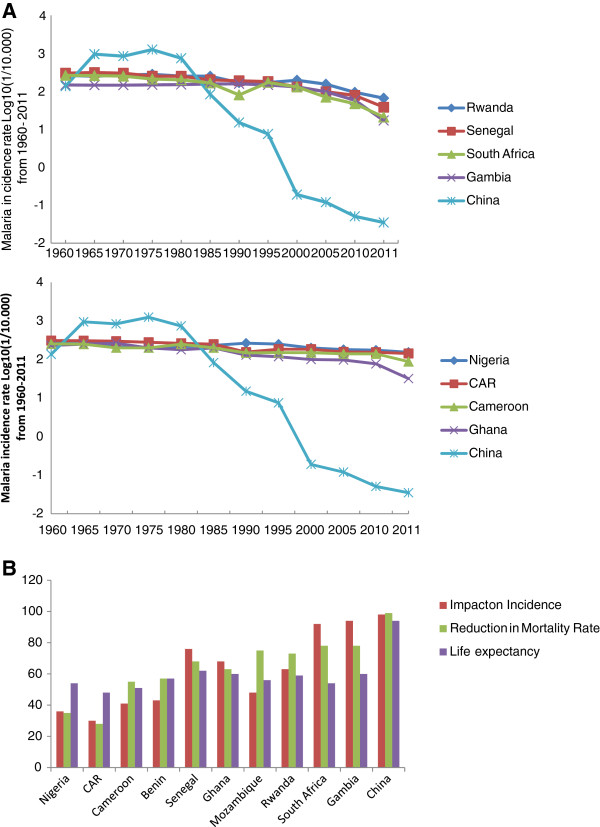
**a: Trend in malaria incidence rate Log**_**10 **_**(1/10.000) in selected African countries and P.R. China from 1960–2011: **(Figure i: Substantial Scaling Up Impact on malaria incidence in P.R. China and some African countries and Figure ii: Scaling up impact on malaria incidence in P.R. China and low/moderate outcomes in some African countries). **b**: Overall Scaling up Impact on incidence and mortality rate, and life expectancy in selected African countries and P.R. China in 2011. (CAR: Central Africa Republic).

At the same time, the public health burden of malaria has continued to increase in most African countries due to poor coverage and accessibility to the needed population in remote areas, weaker health system and importantly the serious threat of increasing antimalarial drug and insecticide resistance, as well as an uncoordinated approach at national and regional levels since 1985 [35,39-41]. The goals oriented interventions are urgently needed by African countries especially learning from those have successfully health policies coupled with the sustained programmes, interventions with significant reduction of the malaria burden through national wide coverage of malaria control measures and has been appraised such as South Africa, Zanzibar, Gambia, Senegal, Ethiopia, Rwanda, Tanzania and Mozambique [Please see Additional file 2[1,2,20-27,30,31]. However, the scaling up impact has not been the same in all African countries, such as The Democratic Republic of Congo (DRC) and Nigeria with a persistent burden of the disease [Figures 1a &1b].

**Figure 2:**
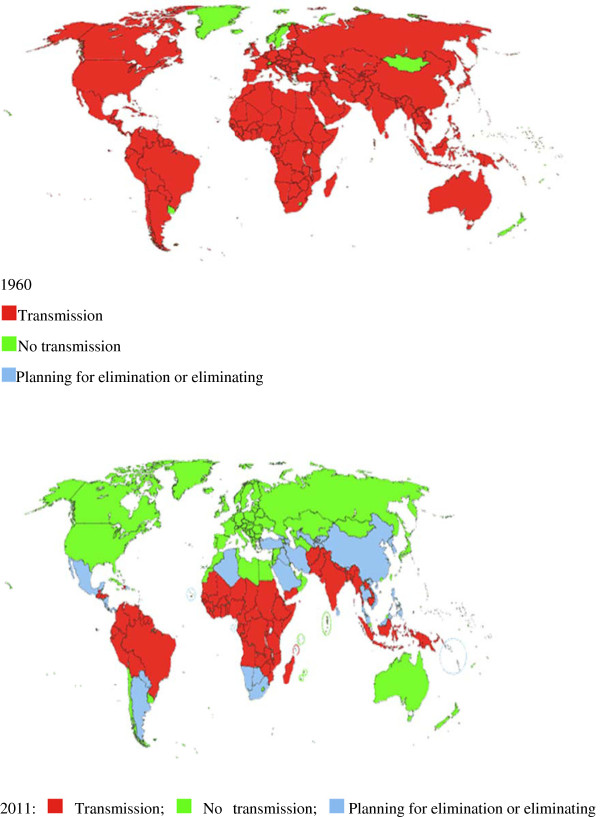
**Malaria distribution worldwide: 1960 and 2011. **1960: (Red square) Transmission, (Yellow-green square) No transmission, (light-blue square) Planning for elimination or eliminating. 2011: (Red square) Transmission; (Yellow-green square) No transmission; (light-blue square) Planning for elimination or eliminating. Source: Malaria Elimination Initiative (2011), UCSF Global Health Group.

The Chinese government’s commitment and intensive interventions towards malaria control and elimination have been enhanced by the GFMAT, Round 1–6 and national strategic applications from 2002–2012, decentralization of Center for Disease Control and Prevention at all levels nationwide since 2000, integrated healthcare systems by broadening health financing options, improving functionality of the National Ministry of Health, improving performance, strengthening case reporting and surveillance systems in rural areas, use of IRS coupled with environmental management to reduce vectors breeding in localized hotspots such as in Tibet, Henan, Hubei, Jiangsu, Guizhou and Yunnan provinces, staff incentives and competition and working with multi-stakeholders, research institutions and private sectors. Consequently, an unprecedented fall of prevalence rate from 0.19/10,000 in 2000 to 0.035/10,000 in 2011, with increasing health system decentralization and delivery, capacity building and life expectancy of plus 30 years operating through an efficient information system network nationwide on malaria surveillance reporting coverage of 97.4% [14,27,35-42]. As a result of scaling up impact, the Chinese government in 2010 launched the National Action Plan for Malaria Elimination till 2020 with the National Guidelines on Malaria Surveillance and Epidemic Response in alliance with efforts in strengthening health system and capacity building in remote areas by improving investment for malaria control and elimination as well as regional collaboration on networks. Similarly, varied degrees of laudable achievements have been made in some proactive African countries committed to the scaling up of malaria control interventions resulting in a marked reduction of morbidity and mortality rates amongst the risk groups and malaria transmission being more focal, with some areas being relatively free such as South Africa, Zanzibar, Ethiopia, Equatorial Guinea, Sao-Tomé & Principe, Gambia, Senegal, Mozambique, Rwanda, Tanzania and Zambia. In contrast Angola, Cameroon, Gabon, Congo, Benin, Cote d’Ivoire and Somalia which are still having substantial risk of malaria endemicity. The DRC and Nigeria have made little progress with malaria control intervention programmes possibly due to large population size, landscape, inadequacies in health system and health policy [1,2] [Figure 2.

### Interventional approaches to malaria control towards elimination

Since 1960, malaria control strategies have achieved substantial successes and there are confounding challenges such as the inadequacies in health systems in counties, lack of access to simple and reliable early diagnosis, emergence and spread of multidrug resistant parasites, *Anopheles* vector resistance insecticides, factors related to environment, demographic and socio-economic status. Our findings showed that healthcare systems with efficient National Malaria Control Programmes (NMCP) having adequate national and global support on malaria control using integrated strategy, including existing early diagnosis tools and prompt treatment, combined with vector control, have shown a significant impact on reducing malaria morbidity and mortality rates.

The strategic approaches on malaria control towards elimination varied from country to country and within settings, and depended greatly on political commitment and financial potentials. These approaches have evolved: (1) modeling through estimation incidence, prevalence and to generate risk maps for all of the world's malaria endemic regions; (2) prevention through the interruption of transmission of the parasite from mosquito vector to humans (and from humans to mosquitoes) and prompt case detection; and (3) management to reduce the incidence and prevalence of malaria infections including severe cases in humans. Knowing the burden of malaria in any country is an essential component of public health planning and accurately estimating the global burden is essential to monitor progress towards the United Nations.

#### Modeling approaches to malaria control

Estimates based on mathematical and statistical methods are used to classify malaria risk into low to high-transmission, incidence, derive the incidence and prevalence rates, cost- effectiveness, time trends and funding-research requirements from malaria epidemiology or empirical data [42]. But each evaluation model has its strengths and weaknesses as well as to highlight areas that need to be improved to provide better assessments and accurate epidemiological data for malaria control and elimination [43,44]. For example in 2000, it was estimated that total of 225 million malaria cases, in the 99 countries malaria endemic countries - the majority of cases (78%) were in African region, followed by the Southeast Asian (15%) and Eastern Mediterranean regions [45]. In Africa, there were 214 cases per 1,000 population, compared with 23 per 1,000 in the Eastern Mediterranean region, and 19 per 1,000 in the Southeast Asia region [46]. Sixteen countries accounted for 80% of all estimated cases globally. The estimate of malaria case incidence for the African region is 176 (110–248) million cases, 261 (241–301) million *P. falciparum* cases in 2007 and 214 million for year 2011 [47,48]. Nowadays, the best assessment of malaria burden and trends must rely on a combination of surveillance and survey data. In recent years, mathematical and statistical models have been used extensively in forecasting of incidence and mortality rates, socio-economic implications in both Africa and Asia, in increasing stakeholder awareness on the disease burden and in estimating the cost (investments and cost effectiveness) in control interventions based on spatio-temporal, ecological and climatic risk factor modeling as well as in assessing the impact of interventions and challenges [49-53].

#### Preventive measures against malaria control

These are measures involving vector control interventions aimed at reducing transmission and thus decrease the incidence and prevalence of parasite infection and clinical malaria. Prevention with intermittent preventive treatment for pregnant women reduces the impact of placental malaria infection and maternal malaria-associated anemia. Early and effective case management of malaria will shorten its duration and prevent complications and most deaths from malaria [54]. Over the years, the preventive measures have been very effective strategies in protecting the most vulnerable groups against vector contact and progression of the infection. The two most powerful and most broadly applied interventions are LLINs [55-58] and indoor residual sprays (IRS) [59]. At the same time, behaviour change interventions including information, education, communication (IEC) campaigns and post-distribution are also strongly recommended [31,55,56]. These interventions act by reducing the lifespan of female mosquitoes and by reducing human-vector contact. In some specific settings and circumstances, these core interventions may be complemented by other appropriate and highly practical effective methods, such as larval source control including environmental management. However, larval control is appropriate and advisable only in a minority of settings, where mosquito breeding sites are few, fixed and easy to indentify, to map and to treat; in other circumstances, it is very difficult to find a sufficiently high proportion of the breeding sites within the fight range of the vector [60].

Malaria vector control, with LLIN, IRS or other interventions, is reported to be only effective if high coverage is achieved and requires timely sustained programme of vector control, and effective delivery operations at national, provincial and district levels [20,22,27,30,31]. In addition, practical experiences in delivery vector control interventions require capacity in monitoring vector-related and operational factors that may compromise the effectiveness of the intervention. However, the spread of insecticide resistance, especially pyrethroid resistance in Africa, is a major threat, requiring a substantial intensification of resistance monitoring within country and across borders as well as research into novel insecticides and larvicides [61,62]. Moreover, malaria vector bionomics and vector distribution maps need to be updated periodically through vector sentinel sites in different ecological and epidemiological risk factors. For example in Kenya, the proportion of malaria outpatient visits declined from 40% in 2000 to 0% by the end of 2006, with the largest decline between 2003 and 2005. Coverage with ITNs in the area is estimated to be 65% higher than that reported on the Africa coast, and 35% of households reported use of some mosquito reduction method, such as environmental management or repellents [20,22,27,30-63]. Similarly, in Rwanda, data from 20 health facilities representing every district in the country showed a decline of more than 50% between 2005 and 2007 in both inpatient and outpatient slide-confirmed malaria cases. Before 2005, the number of cases had been increasing annually, but began to decline shortly before or at the same time as mass distribution of long-lasting insecticidal bed nets and the use of ACTs during 2006–2010 [20-22].

For example, the Zambian NMCP has achieved substantial success in scaling up the use of ITNs with sulphadoxine plus pyrimethamine. ITN ownership increased substantially from 22% in 2004, to 38% in 2006, and 62% in 2008. Between 2006 and 2008, pediatric malaria parasite prevalence declined by 53% and moderate to severe anaemia by 69% [22]. In Central Africa, an urban hospital in Libreville, Gabon reported an 80% decline in the number of children with positive blood smears in the inpatient and outpatient services [22,64,65]. In West Africa, Gambia, where surveillance at five health facilities across the country showed a 50–85% decline in the prevalence of slide-confirmed malaria among outpatients and a 25–90% decline in malaria-related hospital admissions [23 28, 30–48]. The trend persisted over 7 years with an apparent contribution from ITN coverage, which increased three-fold to 49% over the surveillance period. Studies in Africa have shown that ITNs can reduce deaths among under-fives by up to one-third [2,20,27,30,31]. IRS for example, has been a highly effective method of malaria vector control particularly useful for achieving a rapid reduction in transmission during epidemics [54,55]. Reports from Burkina Faso mentioned a three-fold increase in malaria cases at health facilities between 2000 and 2010 in different districts, despite increasing bed net coverage [1,60,65,66]. In P.R. China, the use of LLINs in vector control interventions were integrated in the GFMAT activities in Yunnan, Hainan, and Guizhou provinces, and IRS was used in localized foci of outbreaks in some endemic provinces with a significant reduction of vectorial density to over one percent by 2010. Efforts are now devoted in combating imported malaria, resistance monitoring and containment programs in the Great Mekong region and surveillance along the Three Gorges areas of P.R. China [14-28,30-67]. The IPTp users were documented in most countries for pregnant women living in a high transmission setting receiving at least 2 doses of an appropriate anti-malarial drug during pregnancy as well as non-immune travelers [68]. Other targeted approaches to vector control such as larviciding, environmental management, community education and mobilization are applied wherever appropriate based on scientific evidence. Recently, the applications and uses of Geographic Information Systems (GIS) and Remotes Sensing (RS) have been applied in mapping of the spatio-temporal risk factors of malaria in order to predict the impact of control interventions, possible outbreaks and monitor the vectorial density in any given areas [69-72].

#### Management approaches to malaria control towards elimination

**Figure 3:**
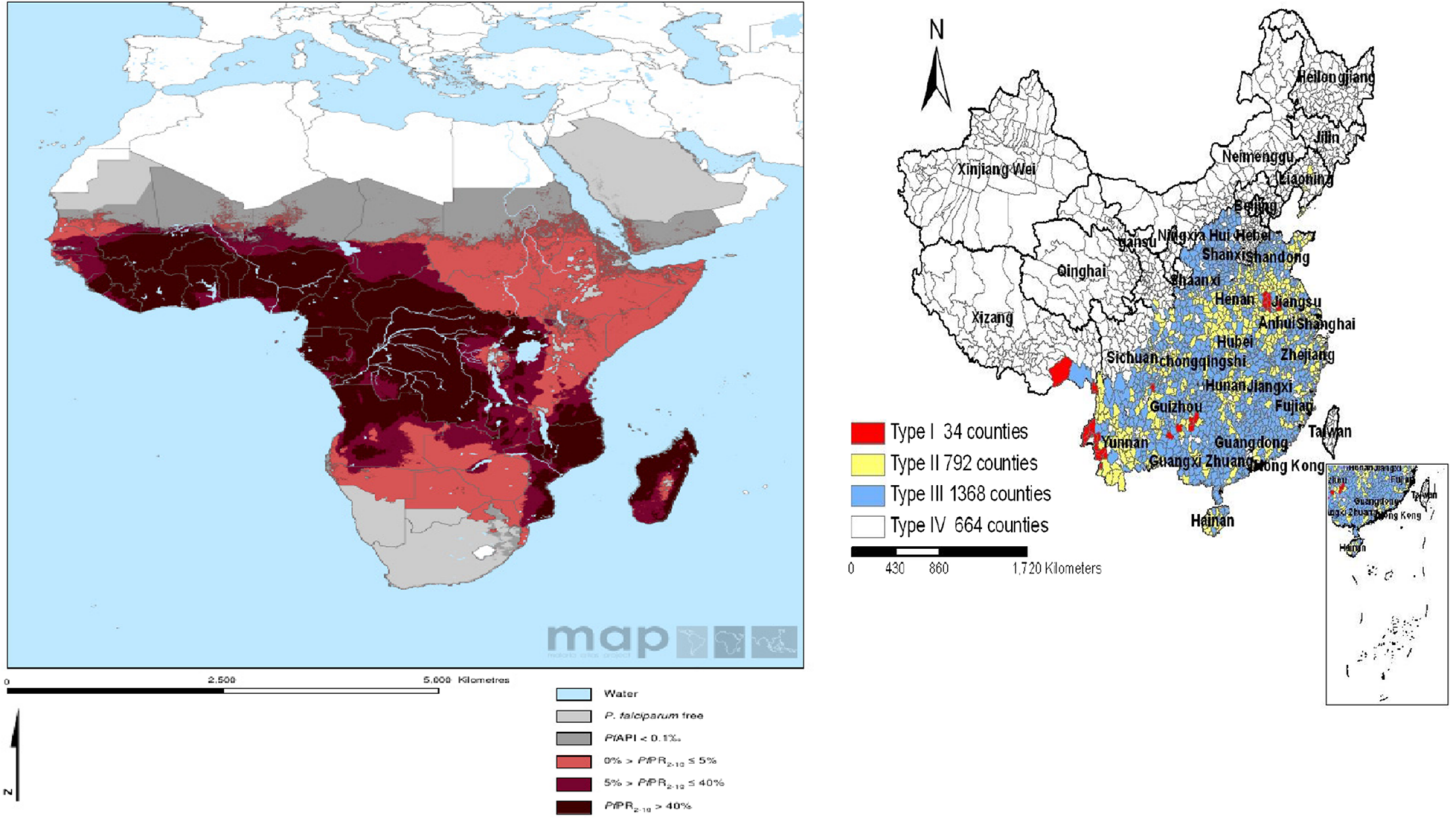
Malaria parasite endemicity in Africa and P.R. China, 2011.

Effective case management using both preventive and curative stage specific antimalarial drugs to all individuals living in malaria-endemic areas through detection of and response to malaria, epidemics through regular disease surveillance, malaria early warning systems, and adequate preparedness plans of action to ensure IRS, ITNs and antimalarial drugs are rapidly deployed when needed. Case management has been achieved over the years both through IPTp in pregnancy and infants at risk of *P. falciparum* infection in countries in Sub-Saharan Africa and radical treatment with stage specific monotherapy or combination antimalarial drugs. Our findings documented that since the early 1960s, the deployment of chloroquine and sulphadoxine-pyrimethamine as drugs of choice in management of uncomplicated cases and quinine in severe cases across SS Africa and P.R. China significantly helped in alleviating malaria mortality rate in Africa and P.R. China. However, the emergence and spread of chloroquine and sulphadoxine-pyrimethamine *P. falciparum* resistance across Africa led to The WHO recommended policy change to ACTs based on proven efficacy of chloroquine and multidrug resistance and tolerability [11,16,30,73-75]. With the past trend of emergence and threat of the spread of antimalarial drug resistance in the Great Mekong region, WHO recommended that in P.R. China, both chloroquine and Dihydroartemisinin plus Piperaquine. But to also include another 3 ACTs recommended in P.R. China’s malaria control guidelines that are first line effective drugs for the treatment of uncomplicated *P. vivax* and *P. falciparum* malaria, which should be combined with a 14-day course of primaquine for the treatment of *P. vivax* malaria in order to prevent relapses (particularly as a component of a pre-elimination or an elimination programme), provided the risks of haemolysis in patients with glucose-6-phosphate dehydrogenase (G6PD) deficiency have been analyzed respectively [1-12,28,29]. Nevertheless, challenges in some African countries include inefficient health systems, poor healthcare service coverage and delivery systems, and drug shortage, counter prescriptions, self medication, fake or counterfeit drugs should be discouraged through health education, pharmaceutical regulations against the decreasing susceptibility to ACTs [76,77]. Hence, the impact of the combined approaches and interventions in malaria control since 1960 to date is summarized below [Figure 1 and Figure 3.

### Sustainability of malaria interventions through health systems strengthening

Strengthening health systems in reaching and maintaining universal coverage both require substantial efforts. Strengthening health systems is not only a malaria specific issue, rather a global development issue deserving the support of the international donor community [55]. The WHO definition of a health system sums up all organizations, institutions, people and resources whose primary purpose is to improve health. It requires adequate staff, funds, information, supplies, transport, logistics, communication, overall guidance and direction. Our findings showed that most African countries had overstretched health systems across malaria-endemic areas, with malaria accounting for an average of 25-35% of all outpatient clinic visits, and 15-35% fatal cases from all hospital admissions, the post independence till 1990s as result of political and financial constraints [45]. Efforts in improving health system strengthening have been set up in most countries in Sub-Saharan Africa and other endemic areas through the RBM and GFMAT support in achieving of MDGs targets Goal 6 and 8, mainly focusing on the most vulnerable malaria-risk population, promoting effective and sustainable malaria control through partnership with governments.

Moreover, health system strengthening in Sub Sahara Africa require the following components: (i) good leadership and governance through strong political commitment backing malaria efforts, clear definition of policy and financing frameworks, leadership and stewardship from national authorities to lead planning efforts and to coordinate all partners; (ii) sustainable financing and social protection through accessibility to adequate and timely resources for activities planned, in ways that ensure populations at risk are covered by the required delivery qualitative interventions without bearing undue personal cost; (iii) efficient and cost effective tools for malaria prevention and case management available for all populations at risk; (iv) good healthcare services delivery should be effective, safe to those that need them, when and where needed, with minimum waste of resources; (v) timely and reliable health information dissemination as well as monitoring and evaluation. Malaria control provides an important platform on which to base additional efforts to strengthen these systems [2-78]. Interestingly, the substantial decline in the last decade in Africa has been as a result of RBM, GFMAT, PMI and other donors supporting, monitoring and forecasting, service delivery by integrating the NMCP and strategies and strengthening health systems through building host country managerial and technical capacity, procurement, quality control, storage, distribution of medicines, and private sector health workers and managers [14,79,80]. In the case of Chinese health system support structure, the Chinese cooperative medical scheme (CMS) was first implemented in rural China in the 1950s, reaching its peak in 1978 by covering 90% of rural residents. This helped reduce Chinese mortality rate from infectious diseases during the 1960s and 1970s. With the collapse of the collective economy in the early 1980s, most villages lost their collective welfare funds, counties then began dropping the program and coverage rates fell sharply from 90% in 1980 to 5% in 1985 [18-27,30-37]. In 2003, to further strengthen the national malaria control programmes, the Chinese government launched the national consolidated medical service (NCMS) aimed at providing health coverage for the nation’s entire rural population and the National Insurance schemes by 2010 [37]. These efforts substantially provide clues that government financial support and decentralized healthcare through Center for Disease Control and Prevention at all levels have been very important, as well as funding from the GFMAT, Round 1, 5, 6 and national strategic applications played a vital role in strengthening, building and sustaining health system efficiency and associated successes that can be derived in malaria control towards elimination. Our findings showed that there is a crucial need for capacity building to the district and local level and also outside the traditional malaria system. In addition, the National Malaria Control Program should be entrusted with the responsibilities in capacity building through training healthcare personnel, strengthening of infrastructure using the best practices in evaluating malaria laboratory diagnosis and proper clinical case management of fever and malaria, in creating a sustainable network of research activities and contribution to malaria control and integrated results of research into policy by linking health workers, researchers and policy-makers; developing and maintaining a viable pharmacovigilance system for anti-malarial drugs and strengthening malaria surveillance activities, establishment and maintenance of a sentinel site surveillance network for the provision of quality data on malaria morbidity and mortality and integrated management of malaria (IMM) through learning, policy and practice health (including malaria) interventions at all levels. In P.R. China, this task has been effectively carried out through the support of Chinese government and GFMAT, Round 1,5, 6 and national strategic applications [81], whereas such training and technical know-how are urgently needed in most endemic areas in SS Africa. Hopefully, the growing P.R. China-Africa Cooperation through the Africa-P.R. China Science and Technology Partnership program should intensify such opportunities by building capacity, supporting cross-bridge between Africa and Chinese scientists and institutions to gain from lessons learnt from P.R. China achievements and successes in moving malaria from epidemic towards malaria elimination [Figure 1a &1b.

## Discussion

### Scaling up impact for universal coverage against malaria

The benefits of malaria control initiated by the Global Action Plan programme towards reducing the burden of the disease was endorsed by RBM, with the main objective of increasing accessibility, availability and affordability of malaria control interventions to the most vulnerable and needy populations living in remote endemic areas and monitoring groups in forest fringe borders areas [30,32,35,40-42].

This review documented that dedicated leadership momentum, proven effective malaria control interventions, and available resources collectively converged to turn the tide against the malaria public health burden and its related effects. These remarkable global achievements in malaria control have been by the dedicated commitment of an array of stakeholders. Similarly, across Africa and P.R. China functional partnerships between government and other key stakeholders, including the academic and educational sector, non-government and community-based organizations, the private sector, religious and faith-based organizations; and multi-/bilateral development partners have proved to be instrumental in malaria control and information dissemination. Based on proven evidence of the effectiveness of the malaria interventions, key determinants of scaling up impact shaped interventional policies and mechanisms of effective deployment of the full package with measurable results in targeted areas [1,20,31,40,41,82,83].

Our finding revealed that from 1960 to 2000, the malaria situation across African countries suffered from a state of dormancy in malaria political commitment and financial support, resulting in an intolerable toll of malaria morbidity and mortality rates as reported in 1998 [62] with some improvements in the course of 2005 and 2010. The alarming scourge was worsened by 2000 by the emergence and spread of *P. falciparum* chloroquine and sulfadoxine-pyrimethamine resistance and *An. gambiense* resistance to insecticides, mainly pyrethroids [55,73,75-84], however substantial improvements in scaling up interventions was accentuated from 2005 – 2010[1-40,83]. In P.R. China the malaria incidence worsened in 1965, 1970 and the early 1980s as a result of severe shortage in health care personnel, the collapse of the cooperative medical system and lack of adequate health policy, were further complicated by major hazards caused by concomitant infectious such as human immunodeficiency virus infection/acquired immunodeficiency syndrome (HIV/AIDS), tuberculosis and schisostomiasis. However, the situation was rapidly addressed with the post 1987 health reforms through expansion and improvement of medical facilities and personnel, decollectivization of agriculture, rural healthcare system, provision of adequate and sustained preventive and curative services, thus resulting in an abrupt decline in malaria burden nationwide [Figure 1a and 1b.

Accordingly, 35 countries in both Africa and South-East Asia are still harboring higher vectorial capacity with high transmission of *P. falciparum* and *P. vivax* malaria which are responsible for the majority of the total deaths worldwide. The major contributors (Nigeria, DRC, Uganda, Ethiopia and Tanzania) account for 50% of global deaths and 47% of cases [1-3], Myanmar, Laos, Cambodia and New Papua Guinea in South East Asia [28]. Accordingly, the benefits of scaling up interventions documented in the last decade as result of increased malaria control interventions varied significantly across Africa, including Eritrea, Zanzibar, Zambia, Gambia and South Africa demonstrated high impact point by showing a marked decrease in morbidity and mortality rates compared to other countries within the Africa continent, but still remained less significant compared to the achievements in P.R. China [Supplement1]. Nigeria, Central Africa Republic (CAR) and DRC had the lowest scaling up impact, calling for the attention of both traditional and also non-traditional donors, in government and the private sector in increasing commitment and funding for accessibility and availability of control interventions to larger populations in remotes areas, and addressing the inadequacies in healthcare service and delivery [Figure 1b. However, achieving the most satisfactory results and maximum health benefits requires a sustained scaling up of integrated malaria control interventions, including prompt and effective case management, use of impregnated mosquito nets and/or indoor spraying with insecticides, intermittent presumptive treatment of most vulnerable groups.

The financing provided for malaria control has enabled endemic countries to greatly increase healthcare systems and delivery capabilities to ITNs, LLINs and case management. The percentage of households owning at least one ITN in sub-Saharan Africa is estimated to have risen from 3% in 2000 to 50% in 2011, the number of rapid diagnostic tests (RDTs) and ACTs procured is increasing from 67% globally in 2005 to 76% in 2010. Reductions in reported malaria cases of more than 50% have been recorded between 2000 and 2010 in 43 out of 99 countries with ongoing transmission, while downward trends of 25%–50% were seen in 8 other countries [1-85]. There is documented substantial progress in use of IPTp and/or ITNs in pregnant women in 28 countries. Similarly, there is marked scaling up coverage progress and substantial beneficial impact across a diverse range of African countries, such as South Africa, Swaziland, Zanzibar, Mozambique, Eritrea, Gambia, Senegal, Rwanda, São Tomé and Príncipe [20,31,35,53-85][Figure 1a &1b. Despite this encouraging progress, our findings showed that there is a high variability and disparity in ITNs/LLINs coverage across African countries over time to endemic population, mainly the vulnerable groups, thus indicating that more efforts are needed before the target of universal access is attained. For example, in Sierra Leone and Togo, the percentage of children under five sleeping under bednets has dropped to < 50% in 2009 after mass distribution campaigns, and was only 25-30% in 2011 [56-65]. The decrease in malaria prevalence is consistent with findings from other countries that high coverage of malaria control interventions (mainly ITNs and ACTs), certainly contributed importantly to the decrease in population infection rate and, consequently, the threat of malaria. The fact in high coverage areas 72% of households with an ITN had at least one person using the net the previous night is encouraging, but also showed that there is still room for improvement. A recent study of 15 standardized national surveys across Africa showed that within ITN owning households, ITN usage by children increases as the number of persons per available net decreases; notably of the 15 countries included in that study [66][Figure 2. It should be noted in achieving maximum impact due to variations between countries in epidemiology and malaria control programs, the appropriate interventions differ by transmission levels, parasite type and vector behavior and delivery strategies need to be adapted to existing control programs and integrated with other disease and development programs by continuously improving health systems to enable malaria control, scaling up and maintaining universal coverage. Particular attention is required to ensure that control interventions reach the most vulnerable populations, and that gender, socio-economic status or geographic location are not barriers to accessibility, availability and affordability.

Furthermore, the review documented that both African countries and a few endemic counties in P.R. China, for example in Yunnan, Hainan and Guizhou provinces, have recorded different degrees of scaling up impact (35-90%) through the national malaria control interventions thus, reducing the rate of morbidity and mortality in children under five years old and pregnancy related effects. By 1959 there were an estimated 1.58 million cases per year. Despite two major outbreaks in the 1960s and then in the 1970s, the country saw a steady decrease in the number of cases, from millions of cases per year to only 29,039 reported cases in 2000 prior the GFMAT. This very encouraging result recorded highlight the evidence that sustained commitment and efforts on preventive interventions and prompt case management are the major driving forces with the resulting benefits of incremental burden reduction of malaria control towards elimination and in achieving the MDGs globally [3,20,32,40,41,58,79,82-85]. For example, promising results were obtained after expanded coverage of malaria interventions, principally LLINs reaching over 60% coverage of populations at risk in both countries and ACTs in Ethiopia and Rwanda; malaria cases in Rwanda decreased by 64% and deaths by 66% between 2005 and 2007 among children under 5 years. And in Ethiopia, cases decreased by 60% and deaths by 51% in the same age group in the health facilities selected for the study [31] [Figure 2.

In P.R. China, the benefits of sustained scaling up interventions on malaria have led to a dramatic reduction of incidence and prevalence rate from 0.19/10,000 in 2000 to 0.035/10,000 in 2011 respectively. The immeasurable benefits include improved health status and life expectancy, increasing productivity, social well being and potential future economic development at national, regional and international levels [28,67,81-86], while most countries in SS Africa would need to toe the same path for a better outcome of investment in controlling malaria. Our findings have also documented that inadequate health systems are one of the main obstacles in scaling up interventions and in securing better health outcomes for malaria, often financial, educational and cultural issues are barriers that need to be addressed in surmounting universal uptake of healthcare services in low resources settings. Since the Abuja declaration was followed by the Roll Back Malaria programme, the Global action plan has contributed immensely to the recent health improvement in African countries with substantial evidence of high achievements through malaria control intervention coverage, especially with ITNs, targeted IRS and use of ACTs to reduce child mortality.

The immense health and economic benefits of scaling up coverage interventions in Africa and P.R. China include reducing morbidity and mortality rates, increasing productivity in the households, community and nationwide, lowering disability adjusted life years, increasing life expectancy, improving healthcare service and delivery, increasing accessibility and availability of infrastructure and adequate equipment and antimalarial drugs, provides additional evidence required to increase long-term national and global political commitment and financial funding, with the ultimate goal that malaria control sustainability leads to elimination and global health. Moreover, control and eventual elimination of human parasitic diseases in the P.R. China requires novel approaches, particularly in the areas of diagnostics, mathematical modeling, monitoring, evaluation, surveillance and public health response [87-89] [Figure 3.

### Challenges in malaria research: progress towards elimination

This review acknowledges that researchers are aware of the constraints in implementing any new program, including political, administrative, financial, operational, social, ecological, and technical considerations. Further operational research challenges will involve dealing with different aspects of sustained malaria control with the aim of bringing different disciplines together to generate new tools and strategies. Some important technical constraints facing malaria include following five approaches: 1) appropriateness and effectiveness of the control interventions against vectors and parasite susceptibility, 2) modeling of risk factors of vectors dynamics, 3) socio-economical and ecological determinants of malaria infections, 4) applications of high throughput technologies in molecular marker identification, genetic diversity studies and searching of malaria potential drugeable target(s) and candidate vaccine(s) using available high throughput technologies and databanks, 5) novel methods of genetic manipulation of *P. falciparum* and *P.vivax*, and metabolomics, and a real time surveillance response system. However, tools alone will not provide all the knowledge needed for sustainable malaria control. Setting malaria control strategies and criteria for monitoring and evaluation of malaria hotspot foci, as well as mapping the risk factors associated with disease using GIS will play a potential role in predicting malaria, epidemics and monitoring control.

It is acknowledged that there were likely to have been imperfections in comparing the whole African continent made up of different countries with different political contexts and health systems as well as different parasites and vector predominance and populations, with P.R. China, one single country with its own internal and cross border challenges, and the inability to assess the progress of ongoing malaria control programmes. Other potential limitations may be in selection bias and misclassification as research publications are not always an accurate mirror of research activities evaluation and policy making. Highly relevant operative research may not be published but are of great value for programmes. Furthermore the extent to which an individual country is associated with a particular publication may vary widely, also in assigning publications with unspecified *Plasmodium* species, publication to one country or subject when multiple countries or subjects are involved. Also, search algorithms targeting the title, keywords and abstract were used to identify and assign publications to multiple countries and subjects.

## Conclusions

The review provides the evidence that supporting national, international political commitment and long-term financial investment in sustaining malaria control towards elimination. Innovative and integrated approaches and interventions can result in significant reductions in *P. falciparum* and *P. vivax* malaria transmission and the associated disease burden in Africa and elsewhere. However, the effectiveness of malaria control interventions may not be uniform across African countries due to the heterogeneous impact on malaria transmission intensity and others related factors. Meanwhile, real time integrated malaria surveillance response system is urgently needed in P.R. China's NMEP against vulnerability and receptivity of *P. vivax*. Moreover, African and Chinese researchers should enhance efficient collaboration and valuable exchanges, preferably with inputs from governments and international institutions/partners in sharing the lessons learnt from Chinese experiences in shifting from malaria control to elimination and in promoting institutions partnerships towards scientific, technological and economical development of new health models targeting the most vulnerable people towards global health.

## Abbreviations

LLIMN: Long-lasting Insecticide-treated Mosquito Nets; LLINs: Long-lasting Insecticide-treated Nets; ACTs: Artemisinin-based Combination Therapies; MOH: Ministry of Health; IRS: Indoor residual spraying; IPTp: Intermittent Preventive Treatment; ITNs: Insecticide-treated mosquito nets; OAU: Organization f Africa Unity; IMM: Integrated Management of Malaria; MDGs: Millennium Development Goals; CCMS: Chinese Cooperative Medical Scheme; NCMS: National Consolidated Medical Service; NMCP: National Malaria Control Program; HMIS: Health Management Information Services; GMEP: Global Malaria Eradication Program; PMI: US President’s Malaria Initiative; GFMAT: Global Fund to Fight AIDS, Tuberculosis and Malaria; WHO: World Health Organization; RBM: Roll Back Malaria; MIM/TDR: Multilateral Initiatives on Malaria / Tropical Diseases and Research; DRC: Democratic Republic of Congo; EU: European Union; US: United States of America; OVID: Originally Publius Ovidius Naso; WHOLIS: World Health Organization’s; MeSH: Medical Subject Heading; UN: United Nations; APMEN: Asian Pacific Malaria Elimination Network; UNICEF: United Nations International Children's Emergency Fund.

## Competing interest

The authors declare that they have no competing interests.

## Authors’ contributions

The review was conceived and designed by ET, papers were retrieved and analyzed by ET. HJ, FH SSZ, LHT provided supportive information and suggestions. ET and AA contributed to drafting the manuscript and all authors gave approval of the final version.

## Supplementary Material

Additional file 1 Multilingual abstracts in the six official working languages of the United Nations. Click here for file

Additional file 2 Trends in the incidence of malaria in selected Africa countries and P.R. China from 1960–2011. Click here for file
