# Supplementary material for: Scaling up impact of malaria control programmes: a tale of events in Sub-Saharan Africa and People’s Republic of China
Source: Infect Dis Poverty. 2012 Nov 1;1:7. doi: 10.1186/2049-9957-1-7 (PMC3710198; doi:10.1186/2049-9957-1-7)
Supplement: Additional file 1 — Multilingual abstracts in the six official working languages of the United Nations. [file 2049-9957-1-7-S1.pdf]

Translation of the abstract into the six official working languages of the United Nations

إرنست تامبو، أحمد أديبوايل أدديجي، فانغ هوانغ، جون هو شن، شوي زن زو، ولينغ هوا تانغ.

خلاصة

هدف هذا الاستعراض الى توفير معلومات منتظمة مع بيّنات علمية حول اتجاهات أحداث الإصابة بوباء الملاريا منذ عام 1960 حتى عام 2011، على أمل أنه سيساعد صنّاع السياسات في اتخاذ قرارات مستنيرة في مسائل الصحة العامة وتصاميم التدخل المتعلقة بمكافحة الملاريا للقضاء عليها في أفريقيا والصين، وذلك من خلال إبراز الإنجازات والتقدّم المحرز والتحدّيات في الأبحاث بهدف الحد من انتشار الملاريا كوباء والقضاء عليه. أظهرت النتائج التي توصّلنا إليها أن التدخلات لمكافحة الملاريا منذ عام 1960 كانت غير مترابطة وغير متناسقة. بشكل لافت وخلال العقد الأخير، تبيّن أن العناصر غير المنتظمة للانخفاض غير المسبوق والكبير في نسب الإصابة بالملاريا والوفيات الناجمة عنها بين المجموعات الأكثر ضعفاً في المناطق هذه حيث يتوطّن هذا الوباء هي متعددة الأوجه، بما في ذلك انتشار الاستعداد السياسي والمادي، الالتزام والتمويل من الحكومات والمانحين الدوليين، توسيع نطاق تغطية التدخلات لمكافحة الملاريا، تحسين نهج النظام الصحي والتزام المستدام لأصحاب المصلحة. أبرز هذا الاستعراض مزايا هذه الإنجازات رغم أنه ما زال هناك الكثير للقيام به في أفريقيا جنوب الصحراء الكبرى. إضافة الى ذلك، فإن استراتيجيات المكافحة المتكاملة المستحدثة من أجل تحويل الملاريا من وباء يجب مكافحته الى القضاء عليه، تتطلب أولويات بحث ثابت من أجل استدامة الوسائل الحالية الأكثر فعالية وتغطية التدخلات، إضافة الى التوصّل الى فهم معمّق أكثر لعلم الأوبئة، الأمراض، ديناميات الناقل، والأوجه الاجتماعية والاقتصادية للمرض. وعلى نحو مهم، ثمة حاجة ماسة لالتزام أصحاب المصلحة السياسي والمادي في إدامة تدخلات مكافحة انتشار الملاريا، والتواصل ودعم العلاقات بين العلماء الأفارقة والصين من جهة وشركائهم الغربيين من جهة أخرى من أجل ترجمة الانجازات والنجاحات الى تدخلات عملية في البلدان حيث الملاريا متوطّنة في أفريقيا وغيرها.

Translated from English version into Arabic by Liliane Hatem, through

TRANSLATORS
WITHOUT BORDERS

疟疾控制规划影响的扩大：撒哈拉以南非洲地区与中国的案例分析

Ernest Tambo, Ahmed Adebowale Adedeji, 黄芳, 陈军虎, 周水森, 汤林华

摘要

本文对非洲地区与中国 1960 年～2011 年的疟疾控制趋势进行了系统综述，科学分析了从疟疾流行到消除的进程中取得的成就、进展以及面临的挑战，为非洲与中国的决策者在从疟疾控制走向消除中的公共卫生问题和干预措施提供决策依据。结果发现，自 1960 年以来，疟疾防控策略与干预措施在不同阶段既未统一也无延续性。但有趣的是，在过去的十年中，即使在疟疾流行的重点地区和脆弱人群中，疟疾的发病率和死亡率也出现了前所未有的下降，这主要归功于政治意愿的加强，资金投入的增多，干预措施的扩大，卫生体制的改善以及多部门的持续支持。虽然疟疾控制已经取得很大成就，但在撒哈拉以南的非洲地区消除疟疾仍有诸多问题尚待解决。而且，在疟疾从流行到消除的进程中的新型综合防控策略需要明确优先研究领域，如现有干预措施及其覆盖的可持续性，疾病发生的流行病学，发病机制，媒介动力学与社会经济因素等方面。重要的是，在疟疾防控策略与干预措施持续扩大的过程中，利益攸关方的政治承诺和资金支持、中非科学家之间及与发达国家伙伴的合作，都迫切需要在非洲及疟疾流行的其它地区尽快地将疟疾控制的成就与成功经验转化为实际的干预措施。

Translated from English version into Chinese by Huang Fang

Extension de l'impact des programmes de contrôle de la malaria : exposé de cas en Afrique sub-saharienne et en Chine

Ernest Tambo, Ahmed Adebowale Adedeji, Fang Huang, Jun-Hu Chen, Shui-Sen Zhou,' Ling-Hua Tang

Extrait

Ce rapport vise à offrir des informations systématiques, prouvées scientifiquement, sur les tendances des cas de malaria entre 1960 et 2011, dans l'espoir que cela aide les responsables politiques à prendre des décisions éclairées sur les problèmes de santé publique et les concepts d'intervention sur le contrôle de la malaria en vue de son élimination tant en Afrique qu'en Chine, en mettant en avant les réussites, les progrès et les défis de la recherche sur les épidémies de malaria. Nos études ont montré que, depuis 1960, les interventions de contrôle de la malaria sont morcelées et loin d'être harmonisées. Il est intéressant de noter que ces dix dernières années, les facteurs déterminants du déclin sans précédent et substantiel des taux de morbidité et de mortalité de la malaria parmi les groupes les plus vulnérables de ces zones endémiques sont très variés, incluant, entre autres, une augmentation de la volonté politique et financière, l'engagement et le financement des gouvernements et de donateurs internationaux, l'extension de la couverture des interventions contre la malaria, l'amélioration des approches des différents systèmes de santé et l'engagement soutenu des parties prenantes. Les avantages de ces avancées sont mis en avant, bien qu'il reste beaucoup à faire en Afrique sub-saharienne. Par ailleurs, les stratégies innovantes de contrôle intégré de la transformation de la malaria d'un stade épidémique au contrôle, puis à l'élimination, exige de solides priorités de recherche, à la fois, en termes d durabilité des outils existants les plus performants et de couverture des interventions, gagnant en outre une compréhension plus approfondie de l'épidémiologie, de la pathogénie, de la dynamique des vecteurs et des aspects socio-économiques de la maladie. Également importants, le besoin d'un engagement politique et financier des parties prenantes pour l'extension des interventions de contrôle de la malaria et de la mise en réseau des scientifiques africains et chinois avec leurs partenaires occidentaux se fait des plus pressants pour la transposition des réalisations et réussites en interventions pratiques au sein de pays africains et autres où la malaria est endémique.

Translated from English version into French by Delphine LE BOULANGER-REID, through

TRANSLATORS
WITHOUT BORDERS

Возрастание положительного влияния программ по контролю малярии: анализ происшествий в Субсахариальной Африке и Китае

Эрнест Тамбо, Ахмед Адебовале Адедежи, Фанг Хуанг, Джун-ху Чен, Шуй-сен Джоу, Линг-хуа Танг

Тезисы

Это исследование предоставляет систематическую информацию и научно подтвержденные свидетельства о тенденциях всплеска малярии с 1960 по 2011 гг. с целью того, чтобы это помогло законодательству принимать обоснованные решения по обеспечению общественного здоровья и планам позитивного вмешатества в контроль малярийных эпидемий для полного искоренения ее в Африке и Китае, подчеркивая достижения, прогресс и трудности в процессе остановки эпидемий малярии. Наши находки показали, что с 1960 г. попытки контроля всплесков малярии были очень нерегулярными и малоэффективными. Интересно, однако, что за последние 10 лет причинные факторы беспрецедентного, но верного уменьшения смертельных и тяжелых случаев малярии среди самых слабых групп эпидемной зоны - оказались самые многогранные: повышение политической и финансовой готовности, обещанное финансирование, предоставленное правительственными и международными организациями-донорами, все более частое вмешательство в распространение малярии, усовершенствование подходов к системе здравоохранения и постоянная поддержка заинтересованных лиц. Положительные результаты этих достижений налицо, однако многое еще предстоит сделать в Субсахариальной Африке. Более того, новые стратегии интегрированного контроля, позволяющие взять под контроль эпидемию малярии и впоследствии полностью искоренить ее, требуют прочных приоритетов исследования как для самодостаточного развития самых эффективных из существующих инструментов, так и для расширения территории охвата медицинского вмешательства, и помимо того, получения более глубоких познаний в эпидемологии, патогенезе, динамике передачи болезней и социоэкономических аспектах болезни. Политическое и финансовое участие членов проекта в увеличении медицинских вмешательств и контроля, развитие научных отношений между африканскими и китайскими учеными, а также их западными партнерами крайне необходимы для превращения теоретических научных достижений в практические медицинские операции по малярийным эпидемиям в Африке и других странах.

Translated from English version into Russian by Elanorielle, through

TRANSLATORS
WITHOUT BORDERS

Incremento en el impacto de los programas de control de malaria: una historia de acontecimientos en África subsahariana y China

Ernest Tambo, Ahmed Adebowale Adedeji, Fang Huang, Jun-Hu Chen, Shui-Sen Zhou,' Ling-Hua Tang

Resumen

El presente estudio pretende brindar información sistemática, basada en pruebas científicas, acerca de la tendencia de los casos de malaria entre 1960 y 2011, con la esperanza de colaborar con el legislador para que tome decisiones informadas en relación a temas de salud pública y diseño de intervenciones para el control de la malaria, con el fin de erradicarla de África y China, mediante destacar los logros, el avance y los desafíos de investigación orientados a la eliminación de esta epidemia. Nuestros hallazgos han demostrado que, a partir de 1960, las intervenciones para el control de la malaria han sido problemáticas y desarmonizadas. En la última década, los factores determinantes de la reducción significativa y sin precedentes en las tasas de morbilidad y mortalidad en la mayoria de los grupos vulnerables de estas áreas endémicas, son multifacéticos e incluyen mayor voluntad política y económica, compromiso y financiamiento de gobiernos y donantes internacionales, incremento en el alcance de las intervenciones de malaria, mejora en el enfoque de los sistemas de salud y el compromiso permanente de los distintos actores. Si bien los beneficios de estos logros son notables, aún queda mucho por hacer en África subsahariana. Asimismo, las innovadoras estrategias integradas dirigidas a la eliminación de la epidemia de malaria exigen que la prioridad en investigación se centre en la sostenibilidad de las más eficaces herramientas a disposición y el alcance de las intervenciones. Resulta igualmente prioritario, lograr una comprensión más profunda acerca de la epidemiología, patogénesis, dinámica de vectores y aspectos socioeconómicos de la enfermedad. El compromiso político y económico de los distintos actores respecto de la sostenibilidad del incremento en las intervenciones de control de malaria, así como el trabajo conjunto entre científicos africanos y chinos y sus colaboradores orientales son vitales para que los logros y éxitos puedan traducirse en intevenciones prácticas en países endémicos de África y el resto del mundo.

Translated from English version into Spanish by Cecilia Klappenbach, through

TRANSLATORS
WITHOUT BORDERS
